# Glucagon-Like Peptide-1 Receptor Agonists and Pay-Per-Click Direct-to-Consumer Advertising

**DOI:** 10.1001/jamanetworkopen.2025.38718

**Published:** 2025-10-31

**Authors:** Daniel Eisenkraft Klein, Marco Zenone, Aaron S. Kesselheim

**Affiliations:** 1 Program On Regulation, Therapeutics, And Law (PORTAL), Division of Pharmacoepidemiology and Pharmacoeconomics, Department of Medicine, Brigham and Women’s Hospital, Boston, Massachusetts; 2 Harvard Medical School, Boston, Massachusetts; 3 University of Ottawa, Ontario, Canada

## Abstract

This cross-sectional study evaluates the use of direct-to-consumer advertising for a glucagon-like peptide-1 receptor agonist (GLP-1RA).

## Introduction

Direct-to-consumer advertising (DTCA) of prescription drugs remains commonplace in the US, accounting for billions of dollars of expenses each year and influencing patients’ and physicians’ perceptions of health issues and treatments.^1^ DTCA has made broad use of pay-per-click (PPC) advertising, in which advertisers target users based on their search queries and online behavior.^2^ With PPC, companies bid on keywords related to their products and pay when someone clicks their advertisements.

Prescription drug promotion is regulated by the Food and Drug Administration (FDA), and manufacturers must follow certain rules, including that advertising be truthful, balanced, and accurate, while disclosing key information.^3^ This study explores how PPC advertising is used in the pharmaceutical context by analyzing a large dataset of keyword ads linked to Ozempic.com to identify key components of digital promotion.

## Methods

To explore the phenomenon of PPC DTCA, we evaluated the case of semaglutide (Ozempic), a GLP-1RA FDA approved for diabetes.^4^ Our cross-sectional study analyzed publicly available data on paid search advertisements for the drug’s website in the US from April 2022 to March 2024 using Semrush, a tool for keyword and PPC analysis.^5^ Semrush offers a reliable tool for examining strategies,^6^ providing estimated data on traffic, identifying paid Google ads, estimating costs, and understanding the keywords advertisers use to reach users.

Our study followed Strengthening the Reporting of Observational Studies in Epidemiology (STROBE) reporting guidelines. Our publicly available data were exempt from ethics review per the Common Rule, and we relied on descriptive analyses conducted in Excel version 16 (Microsoft) and Semrush Guru.

## Results

In the case of Ozempic, an estimated $7.5 million was spent on more than 15 000 paid keywords over 2 years, generating 2.4 million paid visits to the drug’s website. More than 3500 of the keywords (23%) contained no mention of the drug (Table 1), including misspellings of the brand name. Substantial spending covered weight-loss-related terms, including *Ozempic for weight loss* ($302 757) and *Ozempic weight loss* ($188 626), as well as keywords linked to competitors’ drugs such as *Trulicity* ($203 578) and *Mounjaro* ($113 668). More than 600 keywords included *Mounjaro* and more than 1000 keywords included Trulicity, including *Trulicity side effects*, *side effects of Trulicity*, and *Trulicity complaints*.

**Table 1.** Top 20 Keywords That Do Not Contain the Brand Name *Ozempic*, by Traffic Cost, April 2022 to March 2024

Keyword	Total traffic cost, US $
Trulicity	203 578
Mounjaro	113 668
Trulicity side effects	67 820
Tirzepatide	52 042
Foods to avoid with Trulicity	43 274
Mounjaro side effects	26 429
Trulicity dosage	25 180
Semiglutide	24 511
Trulicity complaints	18 852
Side effects of Trulicity	17 352
Trulicity coupon	16 765
Munjaro	16 051
Trulicity dosing	13 849
Semaglutide near me	13 601
Mounjaro cost	10 641
Byetta	10 445
Manjaro drug	10 393
Mounjaro dosage	10 023
Bydureon	9765

In the cohort, 1729 keywords (11%) contained the term *weight* (Table 2). These keywords led to more than 358 000 website visits via advertisements, constituting 14% of paid keyword visits.

**Table 2.** Top Weight-Related Keywords by Traffic, April 2022 to March 2024

Keyword	Paid traffic
Ozempic for weight loss	113 811
Ozempic weight loss	77 218
6 week plan Ozempic weight loss results	14 703
How to get Ozempic for weight loss	13 452
Ozempic weight loss before and after pictures	9608
Trulicity weight loss	8345
Weight loss drug Ozempic	7426
Ozempic and weight loss	6762
Ozempic weight loss side effects	6503
Mounjaro weight loss	5429
Trulicity for weight loss	4413
6 week belly Ozempic weight loss before and after	4177
Mounjaro for weight loss	4066
How does Ozempic work for weight loss	3960
Ozempic dosing for weight loss	3868
Weight loss Ozempic	3415
Weight watchers Ozempic	2654
How to get prescribed Ozempic for weight loss	2594
Ozempic for weight loss side effects	2521

## Discussion

PPC advertising prioritizes company websites over other sources, an outcome that may not be apparent to users expecting search results to reflect informational relevance or objectivity. Manufacturers’ websites inevitably emphasize medications’ benefits, risks, and alternatives in ways designed to drive prescriptions.

Ozempic’s website has been promoted in response to a diverse set of search keywords that include other prescription drugs. PPC advertising may allow companies to promote their drugs for non–FDA-approved uses; for example, individuals searching *weight* keywords may be targeted with an Ozempic paid advertisement. While Ozempic has been shown to help them lose weight, it is not FDA approved for weight loss. This approach may influence consumer behavior by increasing the likelihood that individuals, including those for whom the drug may not be FDA approved or clinically indicated, initiate conversations with their clinicians that lead to a prescription. This single-case analysis limits generalizability, and the relatively modest spending observed may reflect regulatory caution, strategic targeting, or reliance on other digital channels.

Future research should assess whether similar PPC strategies are used across other products or manufacturers. While the FDA provides guidance for manufacturers’ use of social media, this does not cover search engines. In response, the FDA could issue guidelines about or seek to review PPC content to prevent patients from being misled.

## Notes

Supplement. Data Sharing Statement
