# Supplementary material for: Glucagon-Like Peptide-1 Receptor Agonists and Pay-Per-Click Direct-to-Consumer Advertising
Source: JAMA Netw Open. 2025 Oct 31;8(10):e2538718. doi: 10.1001/jamanetworkopen.2025.38718 (PMC12579337; doi:10.1001/jamanetworkopen.2025.38718)
Supplement: Supplement. — Data Sharing Statement [file jamanetwopen-e2538718-s001.pdf]

Data Sharing Statement

Eisenkraft Klein. Glucagon-Like Peptide-1 Receptor Agonists and Pay-Per-Click Direct-to-Consumer Advertising. *JAMA Netw Open*. Published October 31, 2025.
doi:10.1001/jamanetworkopen.2025.38718

Data
Data available: Yes
Data types: Data (not involving human participants)
How to access data: All data provided in attached Excel sheet.
When available: With publication

Supporting Documents
Document types: None

Additional Information
Who can access the data: Publicly available as supplementary materials.
Types of analyses: Any purpose.
Mechanisms of data availability: Any purpose.
